# Supplementary material for: A shallow deep learning approach to classify skin cancer using down-scaling method to minimize time and space complexity
Source: PLoS One. 2022 Aug 4;17(8):e0269826. doi: 10.1371/journal.pone.0269826 (PMC9352099; doi:10.1371/journal.pone.0269826)
Supplement: Attachment — Submitted filename: Response to Reveiwers.docx [file pone.0269826.s001.docx]

Revision required [PONE-D-21-31272]

Original Manuscript EMID: 417fd714d189ce2d

Original Article Title: “A shallow deep learning approach to classify skin cancer using down-scaling method to minimize time and space complexity”

To: PLOS one Editor

Re: Response to reviewers

Dear Editor,

Thank you for allowing a resubmission of our manuscript, with an opportunity to address the reviewers’ comments.

We are uploading (a) our point-by-point response to the comments (below) (response to reviewers), (b) an updated manuscript with yellow highlighting indicating changes, and (c) a clean updated manuscript without highlights (PDF main document).

We look forward to hearing from you and respond to any further queries you may have.

Best regards,

Sami Azam et al.

**Reviewer #1, concern #1:**

**Concerns:** The introduction section is written well, only the author needs to mention the structure of the paper at the end of this section.

**Author response:** First of all thank you very much for the time that you have given for the thorough review of our paper. We highly appreciate that you have pointed out this important aspect of the proposition.

**Author action:** The structure of the paper including all sections is clearly mentioned at the end of the introduction section that can be found on page 7 from lines 129 to 138.

**Reviewer #1, concern #2:**

**Concerns:** Literature Review: Description of related works presented in the Related Works section should be written as a critic point of view. Pros and cons of these methods and how the proposed method has overcome the drawbacks of these methods that also should be explained. Use table to summarizes the literature in terms of the task and the techniques that were employed, include a column that highlights the limitations of the-state-of-the-art approaches.

**Author response:** Thank you for the observation and we are agreeing with the assessment.

**Author action:** We have updated the literature review section per your suggestions, including adding pros and cons of all the mentioned methods and how the proposed method overcomes the drawbacks. A table summarizing the literature review is also presented. All the modifications of the literature review section can be found under section 4, from lines 185 to 298.

**Reviewer #1, concern #3:**

**Concerns:** Figures: Label the x and y axes in the figures: Figure 23 and Figure 25.

**Author response:** We fully appreciate this useful suggestion about the figures and have addressed them accordingly.

**Author action:** Figure 23 is deleted and Table 9 is added in its place that can be found on page 51, on line 896 under section 7.2.

The x and y axes labels are added to Figure 25 and updated to Figure 22.

**Reviewer #1, concern #4:**

**Concerns:** Algorithm: In All the algorithms you must define Input and Output which are missing.

**Author response:** it’s a valuable suggestion indeed. We fully appreciate this useful concern and tried our best to address the issue.

**Author action:** we have rechecked all the algorithms presented in this study and defined the necessary Input and Output.

Algorithm 1: page 24, on line 447 under section 6.1.2.1

Algorithm 2: page 27, on line 496 under section 6.2

Algorithm 3: page 29, on line 510 under section 6.2.1

Algorithm 4: page 33, on line 578 under section 6.2.3

Algorithm 5: page 42, on line 738 under section 6.3.2

**Reviewer #1, concern #5:**

**Concerns:** How the values of parameters and hyper-parameters used in the experiment are determined that needs more discussion.

**Author response:** Thank you for pointing this out and we highly appreciate this feedback. Your observation in this regard is completely understandable. We have added a thorough explanation to address the issue.

**Author action:** Several approaches are employed in this experiment for image pre-processing, segmentation, down-scaling and model training which require different parameters and hyper-parameters. However, all the methods are re-written by adding the values of parameters and hyper-parameters used in the study. A good explanation of determining the suitable parameters for the algorithms of image pre-processing, segmentation is presented. Moreover, Tables of the parameters and hyper-parameters for the four main sections of pre-processing, segmentation and model training can be found in Table 5, 6 and 7 and 15.

Morphological closing: page 22, from line 399 to 422 under section 6.1.1.1. Moreover, a figure is added corresponding to our statements can be found in Fig 6.

Gamma correction: page 25, from line 462 to 477 under section 6.1.2.1 with a newly added figure (Fig 8) and table (Table 4).

Otsu’s Thresholding: page 30-31, from line 523 to 539 under section 6.2.1

Morphological Dilation: page 31, from line 553 to 557 under section 6.2.2

Largest blob: page 34, from line 600 to 614 under section 6.2.4

Hole-Filling: page 36, from line 633 to 643 under section 6.2.5

Bilateral Filter: page 40, from line 698 to 714 under section 6.3.1

Box-Blur: page 42, from line 740 to 743 under section 6.3.2

Moreover, how the suitable parameters and hyper-parameters are chosen for training the model is explained by performing ablation study that can be found on page 52 from line 900 to 915 under section 7.3.

The result of the ablation study is demonstrated in section 9.2, page 63. Here, Table 15 represents the selected parameters and hyper-parameters for the proposed model after conducting ablation study.

**Reviewer #1, concern #6:**

**Concerns:** In order to assert the Robustness of their model, the authors must show model performance as noise rises, i.e., as we move farther away from real data. How they simulate situations where the image quality is corrupted? I suggest that you add some more results on evaluation on noisy data.

**Author response:** This is a fully valuable concern and we think this experiment can have a significant contribution to this field.

**Author action:** After testing out model with Gaussian noise induced dataset; the accuracy did not fall significantly. The explanation of adding noise and performance of the model on noisy data is added on page 81 from lines 1401 to 1413 under section 9.8.

**Reviewer #1, concern #7:**

**Concerns:** What is the limitation of this study?

**Author response:** Thank you for pointing this out. There are certainly some limitations which should be pointed out.

**Author action:** the limitations of this study are added to the main manuscript that can be found in page 86, from lines 1450 to 1455, under section 10.

**Reviewer #1, concern #8:**

**Concerns:** You have used many variables, abbreviations, Nomenclature in the article. From this perspective, an Index of Notations and Abbreviations would be beneficial for a better understanding of the proposed work. Furthermore, please check carefully if all the abbreviations and notations considered in the work are explained for the first time when they are used, even if these are considered trivial by the authors.

**Author response:** Thank you for the observation.

**Author action:** We have added an index of all used variables in our paper and made sure that all the abbreviations are explained for the first time from when they are introduced. Abbreviation indexing can be found on page 3, from lines 36 to 52.

**Reviewer #2, concern #1:**

**Concerns:** Regarding (1), immediately it has to be noted that the initial number of images are not high in the context of training a neural network, which is understandable though because of the relative scarce availability of big skin cancer image databases. However, a further issue can be that usually the inherent diversity of dermoscopic images is not necessarily high. In this way one could expect that a study introducing a new neural network for detecting malign skin lesions would put a sufficiently strong emphasis on augmentation techniques as well as on validation of accuracy scores. Unfortunately, while the paper goes to great length on defining some of the pre-processing techniques applied to the images, when it comes to augmentation, the authors only state they are carrying out contrast and brightness alteration to the original set. It is not even defined, what exactly these transformations are, while justification for not using other common techniques in addition is not made, either. (see section 8.1, lines 649-656). In my opinion this part of the study needs a much greater elaboration. Please insert the math definitions of brightness and contrast transformation and what parameters values were exactly used, and also explore the additional augmentation possibilities.

Furthermore, only a single data split was used throughout the paper, and this split has been done after carrying out augmentation (section 8.2). In this way, it's hard to judge whether the images in the training set sufficiently differ from the ones in the validation and testing sets. In my view, although Fig. 27. Exhibiting the training and validation loss curves would suggest that no overfitting occurs; a more rigorous check has to be carried out to see if this is indeed true. I suggest the authors perform a k-fold cross validation on their data as well as performing the data splits with various ratios and some data splits before doing augmentation. It must be demonstrated that the achieved very high accuracy scores hold up against such shuffling and cross validation.

**Author response:** First of all we like to sincerely appreciate the time you have taken to provide such a constructive and detailed review of our work.

**Author action:**

1^st^ issue

Four photometric transformation techniques are used in this study to increase the diversity and number of image on the dataset. A well and a brief explanation of these techniques with mathematical definitions used parameters values and justification for not employing other augmentation techniques are added to the manuscript that can be found on pages 50 to 52, from lines 838 to 884 under section 7.1.

Performance analysis of geometric and photometric augmentation on our dataset is given on pages 78 to 76, lines 1321 to 1337 under section 9.6.

2^nd^ issue

Rigorous check of overfitting: To carry out a more rigorous check of the performance of our proposed approach, three more experiments are done. At first, we have performed k-fold cross-validation on the datasets before and after applying augmentation. Afterward, both augmented and non-augmented datasets are split using various ratios and results are recorded. A good explanation and results of these experiments can be found on page 79 to page 84, from line 1339 to 1400 under section 9.7.

**Reviewer #2, concern #2:**

**Concerns:** Section 7.1 (Image Pre-Processing): The authors describe various possible algorithms for hair removal and enhancement. They state the algorithms producing the highest PSNR are finally selected to be used in their method. Table 1 displays the measured PSNR values. However, despite that gamma correction provides the second worst value from 4 algorithms for enhancement, finally it has been selected for use as the enhancement algorithm. This is in clear contradiction with the authors' selection principle. Another issue: throughout the paper the various algorithms are described with pseudo-code. This would be fine, but already Algorithm 1 in Section 7.1 is a source of confusion, since it's not an actually used algorithm within the processing method, but rather the selection process for the hair removal and enhancement algorithms. I suggest removing this, and only providing pseudo-code for the actual image processing sub algorithms themselves.

**Author response:** Thank you for your comment. We have looked into the issue and provided more explanation and visualization accordingly.

**Author action:**

1^st^ issue

The process of choosing the algorithm based on the highest PSNR value might not be explained clearly earlier. However, a clear and brief explanation for choosing the gamma correction method is added and table 3 is modified which can be found on pages 18-19, from lines 345 to 354, under section 6.1.

2^nd^ issue

As Algorithm 1 is a source of confusion, which represents only the selection process for the hair removal and enhancement algorithms, it has been removed from page 19 while the pseudo-code for the actual image processing sub algorithms are provided.

**Reviewer #2, concern #3:**

**Concerns:** Line 315, formula (1): Only dilation is defined, the other formulae are missing (compare them with those displayed in Ref. [25])

**Author response:** thank you for pointing this out which is really beneficial to us. We have addressed the issue and added the missing formulas in the manuscript.

**Author action:** The formula for morphological closing is defined as equation (1). Other formulas for erosion and dilation are added in the main manuscript, stated as Formula (2), (3) and (4). All the equations can be found in section 6.1.1.1, pages 20 to 21, from lines 382 to 398.

**Reviewer #2, concern #4:**

**Concerns:** Section 7.1.2.1. Gamma Correction: the authors declare that they have used a gamma value of 1.2, "as higher gamma values were washing out the pixels a lower gamma values caused a loss of important details". My problem with this is that visually this might be true, but did they check if other gamma values were causing also worse performance of their neural network? The other issue is that images taken with various cameras and in different exposure/illumination settings, but even the used color space in the recorded images can also highly influence what gamma correction value would look optimal. Therefore, 1.2 might be suitable for the given set of images the authors used, but it may be completely suboptimal for another image set from a different source. Overall, since various gamma correction values can cause drastically different output, sensitivity of the applied neural network in terms of accuracy must be checked against this parameter. Could you please do such check or in any way justify why a universal value of 1.2 would be suitable for any image sets in which skin cancer lesions should be detected?

**Author response:** A useful and valuable point of concern that needs more explanation and we have addressed it accordingly.

**Author action:** We have included proper justifications for using a universal gamma value, given on pages 25-26, from line 462 to 477 under section 6.1.2.1.

**Reviewer #2, concern #5:**

**Concerns:** Lines 391-393, formulae (4-6): please define what the subindices of 0, 1, 2 for various parameters (,) are, and complete the definition of all parameters in the succeeding text part.

**Author response:** We added the subindices for various parameters and complete definition of all parameters.

**Author action:** The added definitions for formulae (4-6) are now updated to formula (7-9) and proper definitions are provided on page 30 from lines 520 to 524.

**Reviewer #2, concern #6:**

**Concerns:** Line 412, formula (7): this is repetition of formula (1). See my comments above: complete formula (1) and refer back to that one in this subsection

**Author response:** Thank you for pointing this out, we have made the necessary changes to fix it.

**Author action:** formula (2) (updated numbering of formula 1) is completed and updated; formula (7) is removed and referred back to formula (2). It can be found on page 21 from lines 389 to 391 under section 6.1.1.1.

**Reviewer #2, concern #7:**

**Concerns:** Lines 416-417, formula (8): please define what is F and S

**Author response:** Thank you for such a useful suggestion and indeed, we should have checked the manuscript more carefully in the first place.

**Author action:** Formula (8) has been updated to formula (3) and necessary definitions had been added on page 21 from line 392 to 394 under section 6.1.1.1.

**Reviewer #2, concern #8:**

**Concerns:** Line 475, formula (9): what indices k, k-1 signify? define.

**Author response:** thank you for your valuable concern.

**Author action:** Definition of indices of k and k-1 has been added on page 36, lines 627 to 630 under section 6.2.5.

**Reviewer #2, concern #9:**

**Concerns:** Section 7.3. Down-sampling: this section would need some revision. First, the authors state that they are down-sampling the images. However, what is really happening here is an edge-preserving low-pass filtering by using a bilateral filter, followed by a (non-edge preserving) low-pass filtering by a box filter. It is not down-sampling per se, unless the result of the filtering is truly down sampled. I presume that the original resolution of 224x224 pixels is reduced to 112x112, given the radius of the box filter, but it is nowhere stated in the text explicitly. Could you please clarify?

The second issue: it's not completely understandable why both of these filters are needed, if finally the edge-preserving nature of bilateral filtering gets lost anyway. In my view one filter would be enough, it can be bilateral filtering itself with well selected parameters.

Third issue: the authors do not define, what parameter values are exactly used for bilateral filtering ( and ), which can highly influence the output.

Finally, I see the lengthy definition of box filtering in 7.3.2 superfluous, as it's a very basic filter well known in the literature. The subsection could be shortened significantly.

**Author response:** we would like to appreciate the effort that you have given to mark the issues in detail. Your pointed issues have proven to be extremely beneficial in shaping up this research attempt to be even more concrete, focused, and precise.

**Author action:**

1^st^ issue

It was a mistake from our side that the suitable term would be down-scaling, instead of down-sampling. However, what exactly down-scaling means in this study is briefly explained on page 39, from line 667 to 679 under section 6.3.

2^nd^ issue

Justification of using two filters can be found on pages 40-41, from lines 671 to 678. For more justification, the performance of these two algorithms is added in section 9.5.1 and can be found on pages 73-74, from lines 1262 to 1277.

3^rd^ issue

Parameter values used for bilateral filter is provided page 40, from line 699 to 715 under section 6.3.1.

4^th^ issue

Box blur definition is shortened and it is stated on page 41 from line 721 to 753 in section 6.3.2.

**Reviewer #2, concern #10:**

**Concerns:** Lines 578-580, formula (14): the definition of parameters is inconsistent and incomplete: the text refers to O denoting the ground truth image, while the formula has G; m and n are not defined.

**Author response:** We have updated the formula definitions accordingly.

**Author action:** the formula (14) has been updated to formula (15) and the definition has been added on page 44 from line 765 to 767 under section 6.3.2.

**Reviewer #2, concern #11:**

**Concerns:** Line 603, formula (17): what is d_fi and d_d? please define.

**Author response:** The stated concerns regarding definitions of d_fi and d_d have been addressed.

**Author action:** Formula 17 has been updated to formula 18 and a proper definition regarding the concern has been added on page 46, line 789, under section 6.3.2.

**Reviewer #2, concern #12:**

**Concerns:** Section 8.1, Augmentation: see my comments above, this section would need a serious rework, brightness and contrast enhancement algorithms must be mathematically defined and the exact parameter values used for these displayed.

**Author response:** we have already addressed this concern in full and updated the manuscript accordingly.

**Author action:** Explanation of augmentation techniques with mathematical definitions and used parameters are stated in page 50, from line 856 to 875, under section 7.1.

**Reviewer #2, concern #13:**

**Concerns:** Line 743, formula (22): again, parameters are not defined in the text, please complete.

**Author response:** Thank you for pointing out this issue, we added the necessary definitions to make the equation more understandable.

**Author action:** Formula (22) is updated to 25 and stated on page 56; parameters are defined on lines 992 to 996.

**Reviewer #2, concern #14:**

**Concerns:** Line 865, formula (32): this is repetition of formula (17). Please delete this one and refer back to the previous definition.

**Author response:** thank you for pointing this out.

**Author action:** formula (32) has been deleted and referred back to the previous definition found in formula (18) which is an update of formula 17 and proper definition regarding the concern has been added on page 46, line 788, under section 7.3.2.

**Reviewer #2, concern #15:**

**Concerns:** Section 10.6. This is in great part a repetition of section 4 (Literature review). Please compact one of these to avoid unnecessary duplication; I would say preferably Section 10.6. should be the more detailed one, as here the direct comparison is made with the newly introduced method.

**Author response:** This is a very impactful suggestion; the proper modifications had been made accordingly.

**Author action:** Section 10.6 is now updated to section 9.9 and is written in more detail which can be found on pages 82-85, from lines 1415 to 1448.

**Reviewer #2, concern #16:**

**Concerns:** Fig.3.: it is a rather convoluted image, and together with Fig. 4, with which there is a significant overlap, it is hard to follow, what is happening here. I would suggest a clean-up and possibly a well visible dissecting of image processing steps from all else, so that contents of Fig. 3 and Fig. 4. be set apart.

**Author response:** we are fully agreed with the concern and have addressed the issue.

**Author action:** Fig. 3. and 4 have been deleted from the manuscript as all the methods used in this study are described with individual necessary figures of process flow and output**.**

**Reviewer #2, concern #17:**

**Concerns:** Fig. 9.: this flow chart mixes the processing step of gamma correction with finding a good gamma value (that is finally set to 1.2 for all images anyway). There is no need for this, as it adds to the confusion of what is the actual processing and what is selection process for good algorithms and their parameters. Simplify the figure to simply display the gamma correction itself.

**Author response:** We fully agree with your concern and made changes accordingly.

**Author action:** we have modified Fig. 9. as per the suggestion.

**Reviewer #2, concern #18:**

**Concern:** Fig. 19: I see this figure superfluous, it can be deleted

**Author response:** Thanks for pointing this issue and the paper is updated accordingly

**Author action:** Fig. 19 is deleted from the paper.

**Reviewer #2, concern #19:**

**Concern:** Fig. 22: what do the numbers 34567 (number of images??), and 56789 (on top of data augmentation??) signify? These must be incorrect values in my view, please check and/or explain.

**Author response:** This was a fault from our end. We have fixed the figure accordingly.

**Author response:** We have made corrections to Fig.22 and it is updated to Fig. 20.

**Reviewer #2, concern #20:**

**Concerns:** Fig.31. figure legend is missing for the displayed numbers (file sizes in kBytes?)

**Author response:** Fig. 31. is indeed missing legend for the displayed numbers and these numbers are of file sizes in Kbytes.

**Author action:** Fig.31 legend has been added and it is updated to Fig. 28.

**Reviewer #2, concern #21:**

**Concern:** Fig.32 and Fig.34: they are basically showing pairs of numbers compared (those of drive space and training time), which is really not necessary. I suggest to delete these figures.

**Author response:** Concern regarding Fig. 32 and Fig. 34 are very much appreciated.

**Author action:** Fig.32. and Fig. 34 have been deleted.

On top of the above observations and comments, other minor issues, all spelling and grammatical errors pointed out by the reviewers have been corrected. Besides, all the sections and sub-sections are updated based on the newly added experiments.
