# Supplementary material for: A shallow deep learning approach to classify skin cancer using down-scaling method to minimize time and space complexity
Source: PLoS One. 2022 Aug 4;17(8):e0269826. doi: 10.1371/journal.pone.0269826 (PMC9352099; doi:10.1371/journal.pone.0269826)
Supplement: Attachment — Submitted filename: Response to Reveiwers.docx [file pone.0269826.s002.docx]

Original Manuscript EMID: 417fd714d189ce2d

Original Article Title: “A shallow deep learning approach to classify skin cancer using down-scaling method to minimize time and space complexity”

To: PLOS one Editor

Re: Response to reviewers

Dear Editor,

Thank you for allowing a resubmission of our manuscript, with an opportunity to address the reviewers’ comments.

We are uploading (a) our point-by-point response to the comments (below) (response to reviewers), (b) an updated manuscript with yellow highlighting indicating changes, and (c) a clean updated manuscript without highlights.

We look forward to hearing from you and respond to any further queries you may have.

Best regards,

Sami Azam et al.

**Reviewer #2, concern #1:**

**Concerns:** The authors responded to my concern by providing MSE, PSNR and similar metrics for various gamma values, comprised in Table 4 of the revised manuscript. In my opinion this does not provide a proper justification, and the values presented in this table do not make much sense, since it's trivial that 1.2 is the closest gamma value to 1 (that gives an identity transformation) from among the other listed candidates, and as such it is to be expected that it will yield the highest PSNR compared to that when applying the other gamma values. However, if the authors applied a gamma value of 1.05, that would generate an even higher PSNR, as it's even closer to 1.

Therefore, please delete Table 4 and the corresponding text on PSNR values (lines 465-467). It remains acceptable, though, that heuristically a gamma value of 1.2 provided the best performance of the applied model, together with the caveat, that it might not be a universally applicable value to all datasets outside of the ones used in this study.

**Author response:** First of all, thank you very much for the time that you have given for the thorough review of our paper. We highly appreciate that you have pointed out this important aspect of the proposition that we should have addressed earlier.

**Author action:** Table 4 and the corresponding text on PSNR values (lines 465-467) have been deleted.
